# Payment integrity in government programs: Takeaways from incorporating the behavioral sciences in US federal evaluations

**DOI:** 10.1073/pnas.2525993123

**Published:** 2026-07-06

**Authors:** Maya Duru, Hanna Hoover, Heather Barry Kappes, David Schwegman, Brigitte Seim, Mattie Toma, Mary Clair Turner

**Affiliations:** ^a^ School of Information, University of California, Berkeley, CA 94720; ^b^ School of Information, University of Michigan, Ann Arbor, MI 48109; ^c^ Department of Management, London School of Economics and Political Science, London WC2A 2AE, United Kingdom; ^d^Department of Public Administration and Policy, American University, Washington, DC 20016; ^e^ Humphrey School of Public Affairs, University of Minnesota, Minneapolis, MN 55455; ^f^ Warwick Business School, University of Warwick, Coventry CV4 7AL, United Kingdom; ^g^ Better Government Lab, McCourt School of Public Policy, Georgetown University, Washington, DC 20057

**Keywords:** payment integrity, public policy, randomized evaluations, behavioral science

## Abstract

A primary way the US federal government delivers public goods and services is via monetary payments. Ensuring that these payments are calculated accurately, delivered on time, and made to the correct recipients is important for government fiscal health. Inaccurate or delayed payments can weaken public trust in the government and undermine government accountability. In this article, we examine findings from a set of impact evaluations assessing interventions designed to improve payment integrity in US federal programs. The low-cost, evidence-based interventions draw on insights from the social and behavioral sciences and include modification of forms, changes to how and when agencies request information, and altering existing communications. The evaluations were conducted by the US General Services Administration’s Office of Evaluation Sciences in collaboration with agency partners. We extract three takeaways across four representative evaluations. First, the real-world evaluations validate a key implication of the behavioral science literature: interventions that reduce burdens for individuals have small effects that meaningfully improve payment integrity at scale. Second, effects attenuate across interventions and over time, suggesting a need for iterative evaluation. Finally, bureaucratic hurdles and administrative complexity are the main barriers to translating academic insights into real-world government programs. Addressing these challenges will require close collaboration between behavioral scientists and practitioners throughout the intervention design and evaluation process.

The behavioral science movement has spurred efforts among policymakers and academics alike to integrate the use of insights from psychology, economics, and related fields into policy design, across areas including tax administration, delivery of human services programs, and the design of labor programs (1–7). Recent efforts also have sought to understand the reduced efficacy of behavioral science approaches when scaled, including efforts to improve vaccination rates and take-up of social benefits programs (8–11). In this paper, we present findings from four evaluations of behavioral interventions that the US General Services Administration’s Office of Evaluation Sciences (OES) conducted alongside agency partners to improve payment integrity–a longstanding government priority of both the executive and legislative branches relevant to all federal programs where payments are made or services delivered (12). In doing so, we share another approach to integrating social and behavioral science expertise into government programs and highlight hurdles in translating academic findings to policy implementation at scale.

Improper payments, defined as any payment that should not have been made, that was made in an incorrect amount, or did not meet a legal requirement prior to payment, totaled approximately $2.8 trillion since 2003 (12). While improper payments are reported across 16 federal agencies, they tend to be concentrated in the following five programs: Medicare, Medicaid, Unemployment Insurance (UI), Supplemental Security Income (SSI), and the Earned Income Tax Credit (EITC) (13). Ensuring that these payments are calculated accurately, delivered on time, and distributed to the correct recipients is important for the fiscal health of the government as well as ensuring public trust (14, 15).

Much of the focus to date has been on overpayments; according to fiscal year (FY) 2024 agency reports, 84% of the $162 billion in improper payments that year were overpayments (12). Helping beneficiaries receive the correct amount or determine eligibility quickly is desirable, not only from the perspective of maintaining the fiscal health of the federal government but also for helping beneficiaries avoid penalties and sanctions. For example, improper EITC claims can result in taxpayers being banned from claiming the credit for 2 to 10 y, repayment requirements with interest, and potential fraud charges (16). Similarly, overpayments in Unemployment Insurance or Medicaid often require full repayment, sometimes with additional penalties or disqualification from future benefits. However, while discussions of payment integrity tend to emphasize overpayments, the US government definitions of improper payments also include underpayments and those with insufficient documentation (17, 18).

Our paper discusses findings from a portfolio of impact evaluations conducted by government agencies in collaboration with OES aimed at improving this broader class of improper payments by leveraging insights from the social and behavioral sciences. We take stock of lessons learned from four evaluations that are representative of the broader portfolio. We provide perspectives on the feasibility of incorporating evidence-based insights into federal programs as a way to improve payment integrity, assess when these interventions are likely to produce impact, and identify areas where additional evaluation is needed to shed light on the drivers of accuracy and timeliness in federal payments.

The four OES evaluations target individual behavior to address payment integrity, either by reducing underpayments to eligible beneficiaries or by reducing monetary loss from overpayments. The interventions were designed to improve the reporting or eligibility verification process or increase the salience of information and payment processes; they include modification of forms, changes to how and when agencies request information, and alterations of existing communications. For instance, we explore the impact of reminder messages to encourage reporting changes in earnings which might affect the receipt of government benefits, in turn decreasing the rate of overpayments to individual beneficiaries. We also investigate the role of process-focused changes, which, in the cases we evaluate, aim to simplify the process of applying for rental assistance and thereby reduce underpayments to eligible individuals. In all of the evaluations, the interventions are designed to motivate more accurate reporting or the take-up of benefits by eligible (and not ineligible) individuals, ultimately reducing improper payments as a whole.

These evaluations sit within a broader context in which significant efforts have been made to improve payment integrity in the United States in recent years. Several legislative and regulatory acts have been passed, the most recent of which is the Payment Integrity Information Act of 2020, which added greater oversight and consequences for agency noncompliance (18). Other efforts include data matching or data sharing across agencies to confirm eligibility before payments are made (15, 19), improving program design such as by strengthening internal controls (20), payment recovery audits, and training and awareness. However, there still exist numerous obstacles toward eliminating improper payments, including longstanding program complexity (15), limited data quality and interagency data sharing (21), and agency-level resource constraints (13).

While many of the existing efforts to achieve payment integrity have sought to address institutional and structural barriers, there is reason to expect that interventions that address relevant behavioral barriers also might be effective. These behavioral interventions can leverage evidence from the academic literature about how people make decisions and their ability to comply with program requirements. For example, barriers stemming from a lack of knowledge or attention may be addressed by timely communication (22–25). Barriers related to program complexity—such as complex decision rules and compliance requirements, which may result in administrative burdens—may also benefit from increased communication, simplification, or automation of verifying eligibility (26–28). The OES evaluations presented in this paper have been inspired by this behavioral literature.

Still, there are reasons why these behavioral mechanisms may be less effective at improving payment integrity outcomes or fail to scale in a federal government context. For example, we would expect null effects of these interventions if the primary source of errors stems from government oversight or policy design. Similarly, behavioral interventions, which primarily help people follow through on desired actions, might be more effective at preventing unintentional errors than reducing intentional fraud. Relatedly, if an intervention works to reduce overpayments by inducing fear of making errors and the resulting consequences, like penalties or audits, this approach also could have the unintended consequence of discouraging take-up even among eligible beneficiaries.

Moreover, the outcomes measured in the interventions are proxies rather than direct measures of improper payments. For example, when people report higher earnings and thus receive lower benefits, we presume overpayments decrease. Reported earnings are used because direct measures of improper payments require audits, making such data delayed and selectively available. Although proxy measures are useful, they may contribute noise that obscures effects of behavioral interventions. This limitation is most pronounced when the intervention could induce both under- or overreporting of income or sales, making it more difficult to interpret changes as solely reducing overpayments. In these cases, a more cautious interpretation would be to view the proxy measure as an upper bound on the impact on overpayments. In Table 1, we summarize how the four evaluations highlighted in this paper use administrative data as proxy measures of payment integrity and provide an overview of the key behavioral mechanisms that plausibly played a role in driving impact.

**Table 1.** Behavioral contributors to payment integrity and outcome measures

*Evaluation*	*Outreach to Supplemental Security Income recipients* *(SSI Recipients)*	*Outreach to paid tax preparers who made likely errors claiming tax benefits* *(Tax Preparers)*	*Simplifying income eligibility evidence requirements for Emergency Rental Assistance applicants* *(ERA Applicants)*	*Self-attestation prompt for businesses paying an Industrial Funding Fee* *(IFF Vendors)*
Connection to payment integrity	Overpayments are made when beneficiaries do not self-report increases in wages.	Overpayments are made when tax filers overclaim a credit (e.g., by misreporting income) or claim a credit they are ineligible to claim (e.g., by misclaiming a child).	Underpayments are made when eligible beneficiaries are unable to find or provide verification documents, resulting in a denial of benefits.	Underpayments are made when vendors do not pay a large enough fee due to underreporting of sales.
Outcome measure	Self-reported changes to earnings	Tax returns prepared on behalf of clients by tax preparers	Applications, processing time, and approval rate	Vendor-reported quarterly sales
		Probabilistic models capture likely errors on returns from common sources of errors.	Application audit data provide information on regular application reviews for suspected fraud (Kentucky only).	
Outcome measure as a proxy for payment integrity	An increase in self-reporting wages is viewed as a proxy for reducing overpayments.	A reduction in refund amount is viewed as a proxy for reducing overpayments.	An increase in awarded applications is viewed as a proxy for reducing underpayments.	An increase in reported sales is viewed as proxy for reducing vendors’ underpayments of fees to the government.
Plausible mechanisms	Reminder, credibility, novelty and temporal dynamics	Reminder, simplification, simplification, novelty and temporal dynamics	Simplification, self-signaling and commitment	Self-signaling and commitment, credibility, novelty and temporal dynamics

Notes: The IFF Vendor evaluation does not directly address a payment integrity problem where the government makes direct payments or provides services. We include this evaluation in this review, since vendors’ self-reporting of sales to determine their fees owed to the government is a highly relevant context that likely relies on the same behavioral mechanisms behind getting government payment right.

Finally, we may expect muted or null effects in government settings of promising interventions in the academic literature. DellaVigna and Linos (10) conducted a meta-analysis of government behavioral interventions and found that academic studies typically show larger effect sizes than government implementations, primarily because of the publication bias in academic literature. List (29) argues that in more than half the cases where governments try to scale an idea, “it never had voltage to begin with,” often due to false positives in prior research. Thus, successful scaling requires surviving multiple essential and iterative tests rather than simply demonstrating initial effectiveness (30, 31). Consistent with this, Slemrod (32) shows that, depending on the mechanism behind the behavior change, interventions may lose credibility or generate equilibrium effects when implemented economy-wide. Other common hypotheses for why behavioral interventions implemented by the government might fail is that barriers–in this case to program compliance–are too complicated, the message itself is ineffective, or the wrong people are reached (5).

Against this backdrop, the four OES evaluations provide a realistic benchmark for likely effect sizes and success rates at scale for behavioral interventions addressing payment integrity. Looking across the interventions, we find that even small changes, such as reminders, clarifying rules and regulations, or credibly signaling that individuals’ actions are monitored, can have meaningful substantive effects on reducing overpayments, especially when considering the scale at which these programs and interventions are implemented. Complementing these findings, another evaluation highlights how changing an eligibility verification procedure can substantially increase program access, without evidence of increased fraud or time to approval. Moreover, we find that these behavioral interventions are effective when applied across a variety of programs, which serve large and diverse populations, although the effectiveness could diminish over time and with repeat exposure to the same or similar interventions. Finally, we show that behavioral interventions can produce meaningful effects when implemented by government agencies, despite operational barriers and extensive regulatory and legal approval processes that create hurdles for implementation and evaluation.

## The OES Evaluation Project Process and Payment Integrity Portfolio

OES is an interdisciplinary team of social scientists and evaluators that help GSA and other federal agencies build and use evidence, supporting implementation of the Foundations for Evidence-Based Policymaking Act of 2018 (the Evidence Act). OES is a mix of career federal employees and temporary team members who join OES for 1 to 4 y on loan from a home institution in academia, a nonprofit, or other government office at the local, state, or federal level. Noncareer team members are typically brought on as evaluators through the Intergovernmental Personnel Act (IPA) mobility program. They generally hold doctoral degrees in the social sciences and have extensive experience in evaluation methodology.

To design the interventions and evaluations, OES collaborates with agencies across the executive branch to help federal agencies advance their strategic priorities. Since 2015, OES has completed over 120 evaluations with dozens of agencies, from the Department of Health and Human Services to Veterans Affairs to the Small Business Administration. Evaluations often consist of a randomized intervention embedded in a federal government program. Outcomes are typically measured using government administrative data, such as program records, tax returns, or program beneficiary forms.

All OES evaluations follow a detailed and rigorous six-step project process (33). This process was designed to ensure findings are both relevant to agency decision-making and reliable, meaning they can be reproduced and are generated using credible evaluation designs. The project process has evolved over time to encompass more evaluation types and incorporate best practices and practical learnings from working with agencies, with the goal being that they use reliable evidence to inform decision-making.

Projects begin with a structured initiation process to ensure evaluations address high-priority federal goals. Projects are screened for feasibility, including the ability to implement a randomized controlled trial or similarly rigorous design to answer the priority question, reach an adequate sample size to provide reliable evidence, and whether results can be delivered on a timeline that informs real agency decisions. This upfront scoping aligns evaluation questions with operational constraints and policy relevance. Building on this foundation, OES translates social and behavioral science insights into concrete, low-cost interventions that the agency collaborator can implement and evaluate during routine operations. Drawing on peer-reviewed research and practitioner expertise, OES diagnoses behavioral barriers, outlines a clear theory of change, and designs interventions and evaluation strategies that undergo internal peer review. Evaluations are embedded directly into program operations to ensure feasibility at scale. Prior to viewing outcome data, OES registers detailed analysis plans that outline the data collected, intended measurement approaches, and analysis strategies, which are posted publicly on the OES website in accordance with research transparency best practices. Findings—whether they are statistically significant or null effects—are presented in accessible public-facing products that support timely decision making.

While efforts to incorporate behavioral science have often turned to developing toolkits intended for practitioners (34, 4, 35), the OES model brings unique talent in the social and behavioral sciences and evaluation into government to collaborate with agency partners directly on evaluations. This flexible approach allows OES to respond quickly to agency priorities and evidence needs, which are often documented in agencies’ Learning Agendas and Annual Evaluation Plans. For example, one evaluation presented in this paper was conducted to help answer a question in the US Department of Treasury’s Office of Recovery Programs: Economic Recovery Learning Agenda (36). Another evaluation advanced the priorities of the 2020 Cross Agency Priority Goal on Getting Payments Right, which identified behavioral science as an underutilized nondata mitigation strategy for reducing improper payments (37).

## OES Payment Integrity Evaluations

**Fig. 1.**
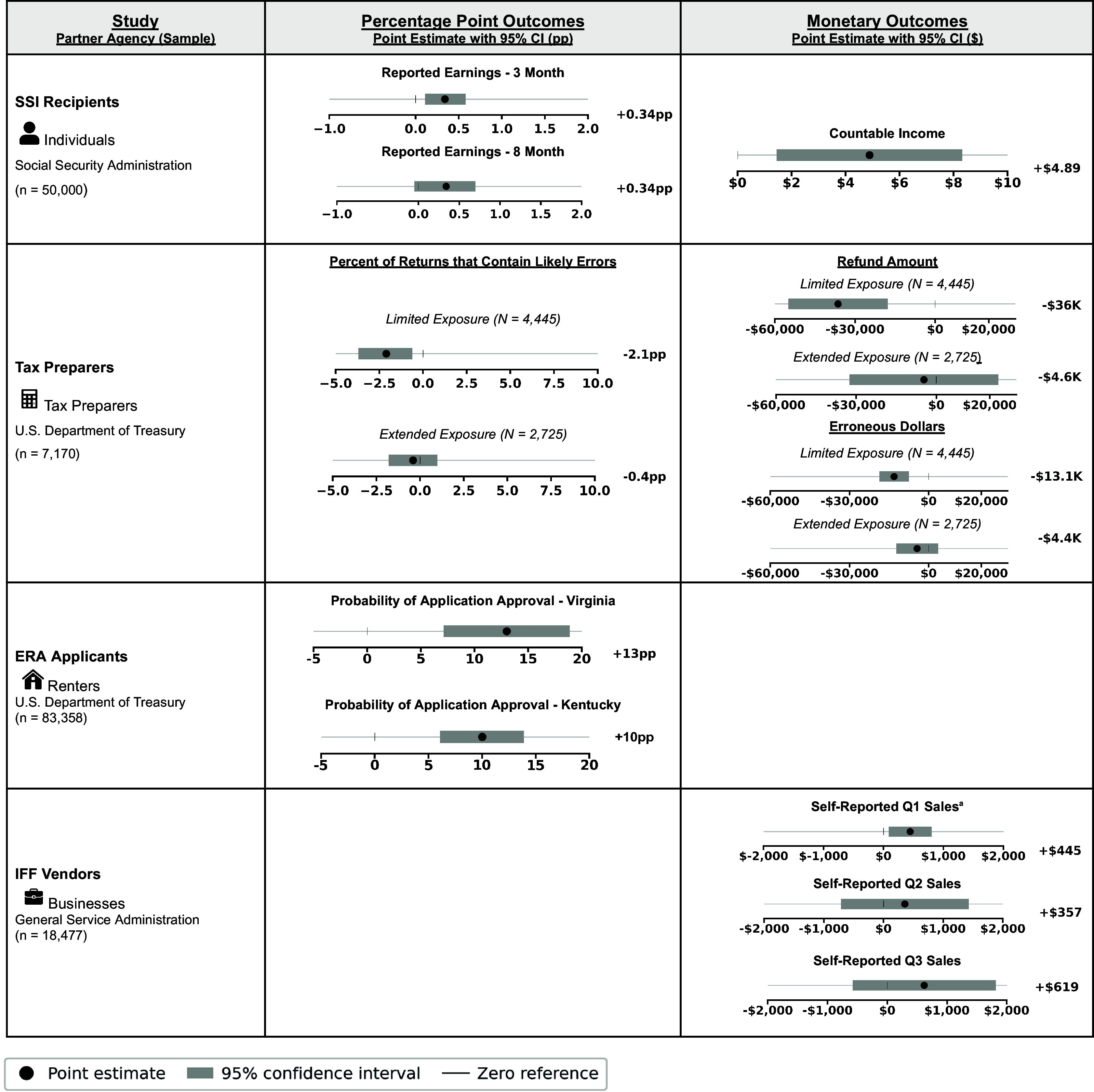
The figure provides the main effect for the prespecified outcome(s) for its respective study, ensuring that results are not the product of post hoc outcome selection, with point estimates (●) and 95% confidence intervals displayed solid gray bars. The vertical line denotes zero. We present the outcomes in percentage point terms on the *Left* and monetary outcomes on the *Right*. Each row corresponds to one study, and it is identified by its sample population (e.g., renters, businesses, etc.) and sample size. The *Top* row presents i) (*Left*) the likelihood of whether SSI Recipients reported any earnings in the prior 3 or 8 mo and ii) (*Right*) increases in countable income reported over the prior 3 mo. The second row presents the change in the likelihood that a tax preparers’ returns contains one or more errors in refundable tax credit claims (for both the Limited and Extended Exposure Tax Preparers groups) on the *Left*. On the *Right*, the third row presents: i) aggregate refund dollars across a preparer’s filed returns (for each Tax Preparers groups) and ii) aggregate dollars erroneously claimed for certain benefits across those returns (for each Tax Preparers group). Please note that the negative estimates in the tax preparer study are indicative of a reduction in overpayments. The third row presents (*Left*) the likelihood of emergency rental assistance application approval (ERA Applicants). The estimates for the ERA studies (Virginia and Kentucky) are drawn directly from *SI Appendix*, Tables S3 and S4 of Simko and Johnson (38). The last row (*Right*) presents self-reported quarterly sales (in dollars) for IFF Vendors. a) The IFF results are based on a median regression.

The four impact evaluations that we highlight here address payment integrity problems in diverse settings and at different stages of the payment process. Two of the evaluations we discuss are published separately (38, 39), while two are discussed here for the first time in peer-reviewed work (40, 41). This section provides an overview of the methods and key findings from these four evaluations. In Fig. 1, we present the estimated treatment effects across these studies. Each row is a separate study, and we present the outcomes expressed in percentage points on the left and the monetary outcomes on the right. We summarize additional project details in *SI Appendix*, Table S1.

To benchmark these studies, we find it helpful to consider the results of DellaVigna and Linos (10), who conducted a meta-analysis of a collection of randomized controlled trials run by nudge units in government, including OES and the Behavioral Insights Team’s North America office, and comparable trials published by academics. These authors found that RCTs published in academic outlets report an average effect size of 8.7 percentage points compared with a smaller average effect size of 1.4 percentage points found by the government nudge units. They attribute the difference to publication bias in academic journals. On balance, the effects in the four evaluations that are the focus of this paper appear more consistent with the meta-analytic estimate from the government trials than from the academic papers, as one would expect.

We also highlight key behavioral mechanisms that motivated the design of the interventions tested in our four evaluations. Notably, these evaluations were designed to test the policy relevance of the intervention, rather than the motivating behavioral mechanism, and so it is possible that other mechanisms are also playing a role in the effects we observe.

### Outreach to Supplemental Security Income Recipients (*SSI Recipients*).

The US Social Security Administration (SSA) administers monthly payments under the SSI program to individuals who are low-income, low-resource, and disabled, blind, or age 65 or older. Zhang et. al (39) presented the findings from an impact evaluation in which SSA randomly assigned 50,000 SSI recipients to one of four versions of a letter (or no letter) reminding them to self-report any changes in earnings that could affect monthly payments or eligibility in the program. Letter variants included, for instance, descriptive information on the number of SSI recipients reporting updated earnings each month or content aimed at increasing the salience of penalties resulting from unreported wages. Three months after the intervention, the reminder letters increased amount of countable income reported from $8.88 reported in the no letter group to $13.77 in the letter groups (*P* < 0.01) and increased the likelihood of reporting any countable earning income from 0.97 percent in the no letter group to 1.30 percent in the letter groups (*P* < 0.01). There was no difference across the four versions of the reminder letters. The study continued to measure reported changes in earnings for 8 mo postintervention. While the effects faded somewhat over time, perhaps because the reminder spurred some recipients who intended to report changes in wages to report these changes more quickly, some impacts persisted through the calendar year.

This intervention was designed to align with the literature suggesting that basic reminders can prompt compliance behavior among individuals who may have limited memory and who temporarily forgot their obligations or opportunities to access benefits (23–25). Behavioral theory indicates that reminders can be especially effective when present focus and procrastination interact with limited memory (22). This is consistent with a broader literature in behavioral economics pointing to the role of reminders in motivating people to convert intentions to action at scale (3, 42, 43). These reminders also provide clear and simple guidance on self-reporting changes in earnings. Finally, the letters might also work through credibly signaling that the agency has personalized information indicating that the recipient is noncompliant in their earnings self-reports (32). Future work can investigate the importance of combining simple and clear reminders with relevant credibility signals.

### Outreach to Paid Tax Preparers Who Made Likely Errors Claiming Tax Benefits (*Tax Preparers*).

Each year, the US Department of Treasury’s Internal Revenue Service (IRS) administers billions of dollars in refundable tax credits, such as the Earned Income Tax Credit (EITC), and the majority of households who claim these benefits use paid tax preparers. In an effort to improve tax compliance in claiming tax benefits, the IRS sends a letter reminding tax preparers of their due diligence requirements to some paid tax preparers who made likely errors in claiming benefits on their clients’ returns in the prior tax season (41). The IRS randomly assigned 7,170 preparers to one of three groups: no letter; the status quo letter, which was sent in prior years; or an updated letter, which was informed by behavioral insights and featured plain language while making salient that information about the likely errors in claiming benefits could be communicated to the preparer’s clients. The letters were sent to two types of preparers: “Limited Exposure Tax Preparers” who had no exposure to IRS education and outreach in the prior 3 y, and “Extended Exposure Tax Preparers” who had recent exposure to IRS treatments in the prior 3 y and also may have received IRS outreach during the filing season. The Limited and Extended Exposure Tax Preparers also differed in their likelihood of making errors and the volume of returns they prepared. In the no letter group, Extended Exposure Tax Preparers were less likely to make errors on a given return than Limited Exposure Tax Preparers (25.2% compared to 31.9%), but Extended Exposure Tax Preparers had a much higher volume of returns (231 returns compared to 121 returns), associated refund amounts ($713,616 compared to $394,528), and dollars claimed with likely errors ($155,071 compared to $108,564).

Sending either letter impacted the returns prepared by Limited Exposure Tax Preparers in the intended direction. In particular, the letter reduced the percent of returns with possible errors in claiming benefits by 2.1 percentage points (*P* < 0.01), reduced the dollar amount of credits claimed with likely errors (by $13,104 per preparer; *P* < 0.01), and also reduced the average refund amount (by $36,351 per preparer; *P* < 0.01). However, the letter had no effect among Extended Exposure Tax Preparers in terms of likelihood of errors (−0.4 percentage points; *P* = 0.56), refund amount (−$4,625; *P* = 0.75), or dollar claimed with likely errors (−$4,364; *P* = 0.25).

The Tax Preparers evaluation shares two key similarities with the SSI Recipients evaluation in interpreting motivating mechanisms. First, the updated letter explicitly communicated that preparers’ clients might also be notified of likely inaccuracies, signaling both increased scrutiny and potential reputational consequences. The fact that the updated letter did not outperform the status quo letter and that sending a letter was effective only among preparers without recent exposure to IRS outreach suggests that the novelty of receiving any letter may play an important role. In particular, receiving this letter for the first time (or first time in the last few years) may have updated preparers’ beliefs about verification probability, i.e., by leveraging credibility signals. Second, the letter served to remind preparers of their obligations and responsibilities, demonstrating that reminders can be effective at improving compliance behaviors among third-party actors who help beneficiaries navigate administrative barriers (26). This finding has practical implications for tax compliance in the United States, since the majority of tax returns that claim refundable tax credits are prepared by paid preparers (44, 45) and tax preparers appear at least as likely as self-filers to claim these benefits in error (46). Together these studies point to the value of low-cost communications used at scale.

### Simplifying Income Eligibility Evidence Requirements for Emergency Rental Assistance Applicants (*ERA Applicants*).

The US Department of Treasury’s Office of Recovery Programs (ORP) provided grants to states to administer the ERA program to reduce housing instability during the COVID-19 pandemic. As part of the ORP economic recovery learning agenda, the office sought to answer the question: “How has the use of promising practices that the Treasury encouraged grantees to adopt affected the equitable distribution of ERA funds and the housing stability of tenants?” (36). To help answer ORP’s high-priority question, Simko and Johnson (38) evaluated interventions introduced in two states, Virginia and Kentucky, aimed at reducing administrative burdens for program applicants. Specifically, both states adapted the ERA program such that the zip code in which someone lived could serve as a fact-specific proxy–that is, a method to verify income eligibility without specific income information for a particular applicant. Using this approach, households in eligible zip codes only needed to include a written attestation of their eligibility in the application, rather than assembling and submitting likely more-burdensome documents to prove their income eligibility for ERA.

Using multiple quasi-experimental approaches that leverage the fact that median area income determines whether a fact-specific proxy could be used to verify ERA eligibility, they found that this approach to simplifying income documentation sped up processing time and increased the probability of approval by 13 percentage points in Virginia (*P* < 0.01, on a base of 33%) and by 10 percentage points in Kentucky (*P* < 0.01, on a base of 43%). The positive treatment effects were consistent across demographic groups. Finally, of particular relevance to payment integrity, there was no sign of an accompanying increase in the detection of fraudulent applications in Kentucky, where data on this outcome was available. This points to the potential value of including self-attestations that an applicant’s income met the program requirements to reduce compliance costs put on potential applicants, at least when other information is readily available to support their attestation. Such self-attestations leverage self-signaling mechanisms, where the act of affirming accuracy makes individuals’ own standards salient to themselves (47).

This intervention was motivated by a literature documenting the impact of administrative burdens–such as complex rules and paperwork, bureaucratic procedures, and long wait times–in decreasing access to and engagement with government programs and services (26–48, 49). In this context, allowing applicants in qualifying zip codes to verify income eligibility through written attestation rather than complex documentation improved access. The power of simplification in government contexts is not a new insight; it is included in a broader academic literature (50, 51) as well as in the Behavioural Insights Team’s canonical “EAST” framework (52). However, such solutions might be overlooked or underestimated by policymakers given the human proclivity to add rather than subtract (53).

### Self-Attestation Prompt for Businesses Paying an Industrial Funding Fee (*IFF Vendors*).

GSA administers an industrial funding fee to federal vendors based on self-reported quarterly sales.^*^ In an effort to identify effective interventions to reduce financial self-reporting errors, GSA randomly assigned 18,477 vendors to an electronic self-attestation prompt–a box in which one types their name to attest that the sales information they are providing is true and accurate at the beginning of the form–or a business-as-usual group which received no prompt (40). The median self-reported sales amount was $445 higher (*P* = 0.01) in the first quarter after the signature box prompt was implemented. The effect was similarly large in magnitude but not statistically significant in longer-run follow-ups ($357 in the second quarter, *P* = 0.51; $619 in the third quarter, *P* = 0.31).^†^

The intervention itself was motivated by Shu et. al (54) and designed to align with the literature demonstrating improved accuracy when anticipated verification and enforcement is perceived to be more likely (55).^‡^ Interventions that signal government monitoring affect behavior by reducing uncertainty costs. They convey the credibility and competence of the government to verify information provided by recipients, provide accurate information to recipients, detect payment errors, and enforce payment integrity. Credibility signaling thereby changes the expected costs of misreporting, by increasing the recipient’s beliefs about the likelihood of detection and consequences, consistent with traditional economic models of crime (56) and tax evasion (57).

On the other hand, the signature box and attestation requirements may also work through self-signaling mechanisms, as in the ERA Applicants study. Interventions that require recipients to certify and attest their submissions heighten the cognitive dissonance that comes with dishonesty, thereby reducing the psychological burden of honesty. In the administrative burdens framework, psychological costs are one type of administrative costs that hinder take-up of benefits (26). This psychological mechanism could explain why the confirmation prompt had immediate effects on reporting behavior, as vendors became more conscious of their own commitment to accuracy. Future work may seek to disentangle the relative roles the mechanisms play in the payment integrity context.

## Takeaways from Low-Cost, Evidence-Based, Real-World Interventions to Improve Payment Integrity

Drawing on this unique portfolio of low-cost, evidence-based, real-world interventions, we identify three actionable takeaways regarding how to improve payment integrity in government programs, as well as evaluate these improvements.

### Behavioral Interventions can have Small Effects on Payment Integrity Outcomes That are Meaningful at Scale.

Recent efforts to understand how promising interventions in specific settings translate at scale–and how different messaging and framing can improve effectiveness–have informed our understanding of the limits and benefits of policy-relevant behavioral interventions. Overall these meta-analyses and megastudies tend to find small statistically significant effects, which are muted compared to similar interventions implemented locally or in academic settings (10, 11, 58). Publication bias contributes to the more muted effects in government settings than academic ones (10). Drawing from the transparency principle in the OES evaluation process, one way academics could help ensure that the best ideas get used in government settings is by making the results of their studies available regardless of the result and registering analysis plans that help to limit reporting of false positives. Providing additional details on interventions also would help make ideas more concrete and easier for government programs to translate behavioral mechanisms to interventions implemented at scale (see for example, refs. 59, 60).

Despite these barriers, a consistent finding in the academic literature is that small impacts can still be cost-effective and meaningful when implemented at scale (5, 61, 62). The OES evaluations under review suggest this finding extends to the application of behavioral interventions to improve payment integrity. The IFF Vendors evaluation, where vendors self-reported higher sales after encountering a confirmation prompt, generated an additional $1.59 million in revenue in a single quarter. In the SSI Recipients evaluations, where reminder letters increased timely reporting rates among recipients, the intervention generated approximately $5.91 in reduced overpayments for every dollar spent by the SSA. Similarly, the tax preparer evaluation showed that sending letters reduced errors and generated an estimated $129 million in cost savings during the 2021 filing season. In the ERA evaluation in Kentucky, reducing compliance costs for some applicants increased approval rates, potentially approving up to 9,500 additional applications, without increasing the number of applications or number of fraud investigations. In brief, these evaluations reveal the merit of simple tweaks to programs and the cumulative effect of small shifts in behavior when samples or program outlays are large.

A related finding is that communications interventions drawing on multiple behavioral insights did not outperform communications drawing on fewer insights. In the Tax Preparer evaluation, the modified behaviorally informed version of the letter—which drew on insights from the behavioral literature pointing to the value of language simplification, consequence salience, and social signaling (2, 63)—produced results that were statistically indistinguishable from the standard letter (which already used many best practices). Similarly, the SSI Recipients evaluation tested different letter variations incorporating social information, penalty salience, and both social information and penalty salience, yet found no meaningful differences in effectiveness across these versions or in comparison to a basic letter. These behaviorally informed variants showed no evidence of additional impact; merely sending any communication was enough to see an effect. Together, the null additional impacts of behaviorally informed language and the consistent effects of providing simple and timely information may hint at limited attention by recipients to nuances of communications they are sent. Perhaps receiving any letter works as a reminder, but most letters are not read carefully enough to allow for noticeable effects of relatively subtle linguistic nuance? These findings from the OES portfolio align with existing literature showing that outreach consistently increases take-up of government benefits (5) and tax compliance (32), while efforts to use theory to inform message content to bolster the effectiveness of the outreach suggest that the relative effects of variations in behaviorally informed messages may not be first-order in driving policy change (11, 64, 65). Turning focus to more practical considerations in the design of communications, for example keeping messages short and making it easy to reply, may show more promise for incrementally improving government communications (66).

In contrast, the intervention that simplified the underlying payment process rather than only the clarity of the communication about the process was particularly effective: The ERA evaluations’ use of location to proxy for income eligibility worked because they reduced complex documentation requirements to simple attestation. This change can be seen as removing some of the “sludge” that could prevent eligible people from accessing benefits to which they are entitled (67). Looking across this set of OES evaluations raises the possibility that larger effects may stem not merely from simplifying the information provided, but from modifying a core process itself (as opposed to communicating with people about how or why to engage with that process). Such process-focused changes may move closer to the “s-frame” (i.e., focus on the systems in which people behave) rather than “i-frame” (i.e., focus on shaping the individual’s behavior) (68); see other examples of process-focused changes in Hallsworth (69). In the administrative burden framework, process-focused changes come closer to addressing compliance costs, rather than information costs (26).

### Effects Attenuate Across Interventions and Over Time, Suggesting a Need for Iterative Evaluation.

A crucial finding observed across multiple evaluations is that intervention effects often waned over time and may be smaller for those already exposed to similar interventions. In the SSI Recipients evaluation, for instance, although persistent impacts of the reminder letter were observed, these effects also faded somewhat over time. The reminder letter might work in part by changing when recipients self-report changes to their income, but not necessarily the level of reporting overall. Although inducing earlier action can result in cost savings, impacts may wane as those who did not receive reminders “catch up” over time. Such effects are one reason it is important to consider the temporal dynamics of an intervention.

Turning to settings where individuals had repeat exposure to behavioral interventions, we posit that novelty effects might be one mechanism that explain attenuation. For example, in the IFF Vendors evaluation, the addition of the self-attestation prompt induced significant effects in the first quarter that became insignificant in subsequent periods. In those subsequent periods, the magnitude of the difference between intervention and business-as-usual groups remained similar (sometimes larger), but the confidence intervals were wider and did not exclude zero, suggesting that the lack of statistical significance was driven by increased variability in the outcome measure. Perhaps when attesting honesty lost its novelty, there may have been more variable individual responses to the intervention.

Similarly, in the Tax Preparers evaluation, reminder letters were effective at improving tax compliance outcomes for tax preparers who had not received recent outreach, but not for those who continued to make errors after receiving outreach in the prior 3 y. One interpretation of this finding is that additional communications have diminishing returns. A new version of the letter may not have been sufficient to induce changes in tax compliance behavior among the preparers with extended exposure to IRS outreach.

These patterns over time and across interventions raise important questions about the sustainability of behavioral interventions and whether their effectiveness can depend on maintaining an element of surprise or novelty. Future evaluations should explicitly test whether rotating intervention designs or varying communication timing can maintain effectiveness over longer periods. Even if rotating intervention designs is infeasible, these findings point to the importance of iterative testing and repeated measurement over time to inform whether an intervention remains effective as recipients become aware of an intervention or policy or the beliefs about detection may change. Academics may have more flexibility to conduct these types of evaluations, especially when evidence is limited or mixed and, thus, not ready to be implemented on a national scale. In turn, government agencies could use findings from academic-led evaluations to identify the most promising data-informed solutions to implement and, ideally, continue to evaluate their effectiveness when taken to scale (70, 71).

### Bureaucratic Hurdles and Administrative Complexity are Key Barriers to Translating Academic Insights Into Real-World Government Programs.

The behavioral sciences literature is a valuable source for designing promising payment integrity interventions. However, the OES payment integrity portfolio highlights challenges in translating academic insights to large-scale government operations. The gap between laboratory findings and field implementation suggests that context, scale, and implementation constraints may substantially moderate the effectiveness of theoretically grounded interventions.

While behavioral interventions are often marketed as “low-cost” solutions, the OES payment integrity portfolio reveals significant variation in implementation complexity that does not always align with effect sizes. Even seemingly simple communication-based interventions often face substantial bureaucratic hurdles and logistical barriers to implement successfully at scale. The IRS letter modifications in the Tax Preparers evaluation required extensive approval processes and legal review, while the multiple letter variants in the SSI Recipients evaluation demanded significant coordination across agencies and offices. These reviews are necessary to ensure government communications include accurate (often personalized) information, are delivered to the correct person, and received in a timely manner to allow recipients to take action. Together these reviews help ensure recipient trust, but they can create challenges for agencies that attempt to implement process improvements before key deadlines (such as the start of the tax filing season), let alone embed impact evaluation into program implementation to learn what changes are effective. DellaVigna et al. (72) have shown that such frictions can even limit the ultimate adoption of evidence-based policy insights, through a process of “organizational inertia.”

Conversely, the ERA Applicants evaluations present a different implementation challenge. These structural changes showed promising effects but could not be evaluated through randomized controlled trials, as government partners were understandably reluctant to randomly assign citizens to different benefit eligibility processes. Instead, these evaluations relied on quasi-experimental methods to evaluate efficacy. As such, these evaluations demonstrate a valuable alternative model where the federal government can systematically collect and analyze evidence from natural experimentation at the state and local levels, potentially creating pathways to scale successful innovations and for academic researchers to contribute to government evidence-building activities using publicly available data.

By adopting principles from the OES project process, academics conducting these types of evaluations could take steps to ensure their work meets the evidence needs of federal agencies. For example, drawing from the structured initiation process at OES, academics could consult agency learning agendas when deciding which projects to pursue.^§^ During the evaluation process, academics could facilitate continued dialog with federal agencies by sharing analysis plans and preliminary findings, providing federal workers the opportunity to give feedback on the alignment of the evaluation with agency needs.

Perhaps most importantly, academics should strive to make their work easily accessible to government audiences. A lack of time is cited as a key barrier to evidence dissemination in policy organizations (74) and limited bandwidth can make it difficult for policy actors–such as government workers implementing programs–to efficiently leverage evidence from dense or technical academic papers (75). To communicate evidence effectively, academics can aim to speak to policy actors alongside academic audiences, for instance by producing tailored policy briefs that directly highlight key insights or compare the proposed policy intervention to others already funded or under consideration. This is especially important given that many policy actors may not have access to academic journals that often sit behind paywalls. By keeping policy priorities as well as policy actors’ own constraints in accessing and engaging with evidence in mind, academics can tailor their communications to facilitate data-informed decisions.

Another insight from the OES portfolio relates to the difficulty in isolating the mechanisms driving the effects of interventions aimed at improving payment integrity when implementing and evaluating them at scale (76, 77). For instance, it can be impractical to include multiple intervention arms to isolate the particular feature of an intervention that is inducing behavior change. Additionally, when measuring outcomes at scale, evaluations tend to rely on administrative data. This has the benefit of providing objective behavioral indicators (e.g., tax filing, income reporting, benefit claiming). However, these data may not capture the full range of behaviors and intermediate outcomes (e.g., submitting verification documents, time spent engaging) that can help isolate mechanisms, especially when additional data collection is prohibitive due to cost, response rates, or other bureaucratic and ethical considerations (78). Mapping the theory of change at the outset of designing an evaluation can support academics and policymakers in tailoring their design and outcome measures to isolate mechanisms to the extent possible given these feasibility constraints.

Together, this work suggests that evidence-based policymaking and policy implementation must take into account not just expected effects but also the underlying mechanisms and the feasibility of evaluating and scaling up potential interventions. The interventions most amenable to gold-standard evaluation (communications changes) may have relatively small impacts on average, while structural process changes often introduce challenges for embedding randomization into the implementation of the program change, due to a misalignment with program goals or other operational barriers. In sum, “ease of implementation” should be assessed not just on intervention complexity, but on the entire evidence-to-implementation pipeline, including evaluation feasibility, approval processes, and scaling mechanisms.

## Discussion

The evaluations examined in this article illustrate that it is possible to effectively design behavioral interventions to improve the payment integrity of government programs. While moving from controlled academic settings to real-world government interventions can yield more modest effects, the scope of government programs means the aggregate impact of such interventions can still be meaningful. However, effects diminish over time as the novelty of the intervention wears off. They may also diminish when individuals experience several similar interventions: a sort of “intervention fatigue” phenomenon.

We offer three promising directions forward for academics interested in contributing to work on payment integrity. First, existing work–including the evaluations discussed here–has for the most part sidestepped the question of intentionality: to what extent errors in payments are intentional or unintentional, on either the payor or payee side. Intentionality is challenging to identify, particularly given that administrative data on improper payments rarely distinguish between unintentional errors and intentional fraud (32). Nevertheless, given that behavioral science interventions are often designed to help individuals act on their underlying intentions (6, 79), future research that disentangles intentionality may facilitate more targeted policy design.

Second, it is worth noting that behavioral factors are just one of many barriers or facilitators to achieving payment integrity or improving government programs. Even if all behavioral barriers are addressed, there is still room for institutional frictions and agency noncompliance, errors in billing that lead to overpayments, limited data sharing, and other technical issues to play a role. Future research on payment integrity should explore how these other levers can improve payment integrity.

Finaly, in this article, we document both the promise of behavioral interventions to improve payment integrity and the bureaucratic and administrative challenges that emerge in the design and implementation of evaluations of payment integrity interventions outside of academia. We conclude by emphasizing the benefits of the OES model, which navigates these hurdles by bringing academic expertise into government alongside a reliable, rigorous, and transparent OES project process. This process is established yet adaptable, grounded in research best practices as well as attuned to the administrative requirements and coordination necessary in large-scale government programs. Academics outside of OES who wish to support real-world policy evaluation could adopt this model for broader applicability. All elements of the project process are freely available on the OES website, including templates that academics could easily adapt for different partners, policy areas, or research questions. As more academics venture into the world of government evaluation, it is important to compile knowledge about how to structure academic-government partnerships to generate rigorous, replicable, and actionable findings.

## Supplementary Material

Appendix 01 (PDF)

## Data Availability

There are no data underlying this work.

## Footnotes

^*^Note that this evaluation does not directly observe or definitively document payments made by the government, but we expect the behavioral mechanisms underlying self-reports of payments made to be highly related.

^†^The median reported amount in the business-as-usual group is unavailable in report materials.

^‡^We note that Shu et al. (54) was retracted after it formed the basis of this intervention design.

^§^Beginning in FY 2027, these will be referred to as “evidence plans” (73).
