# Supplementary material for: Payment integrity in government programs: Takeaways from incorporating the behavioral sciences in US federal evaluations
Source: Proc Natl Acad Sci U S A. 2026 Jul 6;123(28):e2525993123. doi: 10.1073/pnas.2525993123 (PMC13367774; doi:10.1073/pnas.2525993123)
Supplement: Supplementary file 1 — Appendix 01 (PDF) [file pnas.2525993123.sapp.pdf]

Supporting Information for Payment Integrity in Government Programs: Takeaways from Incorporating the Behavioral Sciences in U.S. Federal Evaluations

Table S1

Table S1. Evaluation summaries

	Outreach to Supplemental Security Income recipients (SSI Recipients)	*Outreach to paid tax preparers who made likely errors claiming tax benefits (Tax Preparers)*	*Simplifying income eligibility evidence requirements for Emergency Rental Assistance applicants (ERA Applicants)*	*Self-attestation prompt for businesses paying an Industrial Funding Fee (IFF Vendors)*
Background				
Federal agency	Social Security Administration	U.S. Department of Treasury	U.S. Department of Treasury	General Services Administration
Office	Office of Retirement and Disability Policy	Internal Revenue Service, Wage & Investment	Office of Recovery Programs	Federal Acquisition Service
Location	National	National	Virginia and Kentucky	National
Evaluation				
Design	Randomized Controlled Trial	Randomized Controlled Trial	Quasi Experimental Design	Randomized Controlled Trial
Sample	SSI Recipients	Tax Preparers	ERA Applicants	IFF Vendors
Sample size	50,000	7,170	83,358	18,477
Subsamples	N/A	Limited Exposure Preparers (N=4,445)<br>Extended Exposure Preparers (N=2,725)	Virginia applicants (N=8,124)<br>Kentucky applicants (N=75,234)	N/A

(continued)

Table S1. Evaluation summaries (continued)

	Outreach to Supplemental Security Income recipients (SSI Recipients)	*Outreach to paid tax preparers who made likely errors claiming tax benefits (Tax Preparers)*	*Simplifying income eligibility evidence requirements for Emergency Rental Assistance applicants (ERA Applicants)*	*Self-attestation prompt for businesses paying an Industrial Funding Fee (IFF Vendors)*
Intervention				
Intervention period	2015	2020-2021	2021-2023	2014-2015
Touchpoint	Letter	Letter	Application change	Form change
Intervention details	Four letters: (a) Basic (no behavioral framing); (b) Social Information; (c) Salience of Penalties or (d) Social Information and Salience of Penalties.	Two pre-filing season letters: a treatment-as-usual or behavioral insights. Extended exposure preparers sent a letter could also receive a phone call or letter during the filing season if they continued to make likely errors.	Written attestation of income eligibility for applicants living in zip codes with below median income, since living in those zip codes provided a proxy for meeting income requirements	Electronic signature box at the top of the form confirming "I promise that the information I am providing is true and accurate"
Status quo (control)	No outreach	No outreach	Provide documents to verify income eligibility	No prompt or electronic signature box
Impact				
Impact at scale	Letters induced 132 additional SSI recipients to report any earning three months post mailing	Letters to Limited Exposure Preparers led to 16,637 additional returns being prepared without likely errors	Written attestation of income eligibility among applicants living in low-income zip codes led to 9,500 more ERA approvals in Kentucky.	The form change increased IFF fees paid by $1.59 million in one quarter.
	Letters reduced overpayments of approximately $5.91 per $1 spent	Letters reduced overpayments by approximately $129 million	The procedure change led to no increases in time to approval, the number of applications, or evidence of fraud.	
